# Supplementary material for: Recursive splicing is a rare event in the mouse brain
Source: PLoS One. 2022 Jan 28;17(1):e0263082. doi: 10.1371/journal.pone.0263082 (PMC8797253; doi:10.1371/journal.pone.0263082)
Supplement: S1 Fig — (A) The mapping statistics and access numbers of RNA-seq data utilized in this study. (B) The loci and numbers of junction reads (#) of the 84 RS site candidates. (C) Snapshots of the junction reads at the Huwe1 and Lmbrd1 RS candidate sites. (D) Heatmap of numbers of RS junction reads at each RS site in different total RNA-seq data sets. A green box indicates that the RS site was identified in that data set. Note, the enrichment of junction reads in whole cell data compared to that in mRNA-seq data is less than 2-fold for Ank3. (E) The loci and read numbers (#) of the top 50 additional RS site candidates when lowering the cutoff of junction read count from 10 to 5. (PDF) [file pone.0263082.s001.pdf]

A

	Exon	Intron	Intron and exon	Intergenic	Totally uniquely mapped reads	NCBI SRA access number
mRNA-seq rep 1	169,199,705	52,536,837	9,942,820	18,955,236	250,634,598	SRR5048041
mRNA-seq rep 2	169,212,702	54,408,558	9,418,272	24,448,180	257,487,712	SRR5048042
Whole cell total RNA-seq rep 1	28,025,020	21,276,188	1,708,014	4,537,328	55,546,550	SRR3679862
Whole cell total RNA-seq rep 2	24,025,585	18,552,805	1,481,557	3,816,417	47,876,364	SRR3679863
Nuclear total RNA-seq rep 1	12,802,711	55,975,659	2,066,992	7,376,058	78,221,420	SRR3679866
Nuclear total RNA-seq rep 2	84,581,864	291,193,877	12,151,804	40,616,263	428,543,808	SRR9202881
Excitatory neurons rep 1	7,493,883	46,181,238	1,421,238	5,474,411	60,570,770	SRR3679830
Excitatory neurons rep 2	7,592,500	58,702,729	1,502,461	7,037,646	74,835,336	SRR3679831
Excitatory neurons rep 3	7,028,945	49,007,305	1,469,493	5,869,141	63,374,884	SRR3679832
Excitatory neurons rep 4	8,665,201	71,350,839	1,771,990	8,080,848	89,868,878	SRR3679833
Inhibitory neurons rep 1	10,620,896	51,820,218	1,846,752	5,923,736	70,211,602	SRR3679842
Inhibitory neurons rep 2	11,526,209	61,516,565	1,991,467	7,354,451	82,388,692	SRR3679843
Inhibitory neurons rep 3	10,531,020	44,429,100	1,591,223	5,326,315	61,877,658	SRR3679844
Inhibitory neurons rep 4	8,673,268	50,820,368	1,494,799	6,176,575	67,165,010	SRR3679845
Female excitatory neurons rep 1	12,995,467	56,964,384	1,920,090	7,096,375	78,976,316	SRR3679869
Female excitatory neurons rep 2	9,548,905	49,872,640	1,579,266	6,385,437	67,386,248	SRR3679870

B

Loci	#	Loci	#	Loci	#	Loci	#	Loci	#
chr9_-_29674405	357	chr16_+_41206970	75	chr3_-_158361563	23	chrX_-_139990713	15	chrX_-_79478788	10
chr1_-_183339388	332	chr9_-_82916025	75	chr5_-_86060897	23	chr13_+_13755115	14	chr8_-_50162064	10
chr9_+_28029505	283	chr7_-_61241446	74	chr2_+_179627792	21	chr7_+_96232097	14	chr16_-_57444992	10
chr16_-_67364249	264	chr9_-_49719366	72	chr2_-_7282579	21	chr3_+_14131308	13	chr4_-_48248658	10
chrX_+_151836220	200	chr11_+_80386178	66	chrX_-_51473340	20	chr13_+_13753787	13		
chr11_-_77601762	197	chr16_+_40655810	64	chr18_-_12893721	19	chr14_+_32217381	12		
chr2_-_60076912	180	chr6_-_102503526	64	chr14_-_123590928	19	chr14_+_76119201	12		
chr14_+_119537648	171	chr9_+_47633924	60	chr7_+_87589088	19	chr1_+_180121414	12		
chr16_-_67142935	170	chr4_+_84984367	59	chrY_+_90808704	19	chr1_+_180121489	12		
chr15_-_73365196	146	chrX_+_112602663	54	chr2_-_39082928	19	chr19_+_47602798	12		
chr16_+_40262266	144	chr2_+_35124192	52	chr7_-_84005996	18	chr19_-_28354760	11		
chr10_+_69595048	143	chr1_-_156893013	45	chr4_-_56904127	18	chr12_+_102967315	11		
chr9_-_16029361	129	chr13_+_13755388	45	chr12_-_67059699	18	chr4_-_25728363	11		
chr9_-_49642259	113	chr13_+_13753651	44	chr3_+_159150264	18	chr8_-_99410596	11		
chr13_+_109333510	101	chr13_+_13754977	37	chr15_-_35088437	18	chr9_-_123653345	11		
chr12_-_104738849	91	chr13_+_109550007	33	chr2_-_136200636	17	chr1_-_123810502	10		
chr16_+_40979498	88	chr6_-_94050767	33	chr1_+_24684097	17	chr2_+_20490509	10		
chr16_-_74151684	83	chr1_-_55016183	30	chr13_+_13753821	17	chr14_-_73297836	10		
chr9_+_72309250	79	chr13_+_13755217	29	chr13_+_13753923	16	chr16_+_6158058	10		
chr6_-_37058429	77	chr13_+_109614417	28	chr6_-_148206211	16	chr12_+_65101518	10		

C

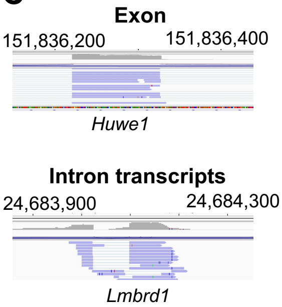

D

	Gene	Whole cell	Nuclear	Excitatory	Inhibitory	Excitatory (female)	Novel
chr9_-_29674405	*Ntm*	24	357	144	113	49	Yes
chr9_+_28029505	*Opcml*	57	283	139	89	55	------
chr16_-_67364249	*Cadm2*	37	264	98	104	49	------
chr10_+_69595048	*Ank3*	14	143	149	97	71	------
chr16_-_67142935	*Cadm2*	31	170	56	60	43	------
chr14_+_119537648	*Hs6st3*	14	171	23	25	29	------
chr9_-_49642259	*Ncam1*	12	113	33	37	18	------
chr13_+_109333510	*Pde4d*	6	101	28	16	20	------
chr16_-_74151684	*Robo2*	4	83	25	28	13	------
chr9_+_47633924	*Cadm1*	10	60	25	21	24	------
chr9_-_49719366	*Ncam1*	6	72	15	22	11	Yes
chr13_+_109614417	*Pde4d*	5	28	23	24	15	------
chr13_+_109550007	*Pde4d*	2	33	5	9	4	Yes
chr16_+_40262266	*Lsamp*	20	144	77	132	31	Yes
chr16_+_40979498	*Lsamp*	12	88	33	47	11	Yes
chr16_+_40655810	*Lsamp*	6	64	19	55	15	Yes
chr16_+_41206970	*Lsamp*	8	75	27	31	7	Yes
chr2_+_179627792	*Cdh4*	0	21	11	13	5	Yes
chrX_-_51473340	*Hs6st2*	3	20	2	1	3	Yes
chr11_-_33718266	*Kcnip1*	1	8	1	70	0	Yes

E

Loci	#	Loci	#
chr7_+_123384756	9	chr9_+_113765922	8
chr2_-_125842760	9	chr14_+_105299009	8
chr9_+_76128920	9	chr6_-_108179985	8
chr1_-_154841063	9	chr13_+_96575819	7
chr11_-_37156217	9	chr1_-_97759169	7
chr10_+_21309092	9	chr1_+_11548381	7
chr2_+_177075009	9	chr1_-_183103753	7
chr1_+_7090857	9	chr9_-_112228936	7
chr11_-_11429127	9	chr11_-_47058958	7
chr11_-_77596357	9	chr1_-_136304718	7
chr13_+_13755081	9	chr2_+_102665261	7
chr8_+_20631694	9	chr4_+_24521096	7
chr1_-_13257060	9	chrX_-_85721958	7
chr8_+_88702858	9	chr2_+_20293447	7
chr8_-_128953205	8	chr3_+_40857854	7
chr2_-_37697912	8	chr2_-_64027968	7
chr14_+_105298953	8	chr11_-_76535247	7
chr11_+_77809797	8	chr12_+_82286608	7
chr5_+_108069164	8	chr8_-_50500205	7
chr7_-_98650773	8	chr2_-_39094563	7
chr16_-_56703624	8	chr14_+_14807267	6
chr13_+_13632543	8	chr14_+_122681487	6
chr17_+_21569014	8	chr15_+_22649279	6
chr11_-_33718266	8	chr4_+_36041544	6
chr6_-_43519253	8	chr7_-_34213648	6

S1 Fig. Novel RS sites. (A) The mapping statistics and access numbers of RNA-seq data utilized in this study. (B) The loci and numbers of junction reads (#) of the 84 RS site candidates. (C) Snapshots of the junction reads at the *Huwe1* and *Lmbrd1* RS candidate sites. (D) Heatmap of numbers of RS junction reads at each RS site in different total RNA-seq data sets. A green box indicates that the RS site was identified in that data set. Note, the enrichment of junction reads in whole cell data compared to that in mRNA-seq data is less than 2-fold for *Ank3*. (E) The loci and read numbers (#) of the top 50 additional RS site candidates when lowering the cutoff of junction read count from 10 to 5.
