# Supplementary material for: Recursive splicing is a rare event in the mouse brain
Source: PLoS One. 2022 Jan 28;17(1):e0263082. doi: 10.1371/journal.pone.0263082 (PMC8797253; doi:10.1371/journal.pone.0263082)
Supplement: S2 Fig — #, number of junction reads. (PDF) [file pone.0263082.s002.pdf]

Loci	#	Motif
5_+_109932925	245	AGGC
16_-_48972609	239	AGGC
6_+_104544118	201	AGGC
7_-_61276541	106	AGGC
5_-_107837447	90	AGGC
11_+_94024915	83	AGGC
7_-_61177531	53	AGGC
2_-_150343156	45	AGGC
6_-_37059005	43	AGGC
11_-_106869109	42	AGGC
6_-_135971965	42	AGGC
13_+_83876759	41	AGGC
16_-_31129626	37	AGGC
16_-_96059674	33	AGGC
16_-_4147165	31	AGGC
12_-_55695897	28	AGGC
2_+_158430383	26	AGGC
11_-_76440037	20	AGGC
18_-_34491113	20	AGGC
1_+_146564463	17	AGGC
1_+_105716092	17	AGGC
15_+_81617839	14	AGGC
6_-_128196834	13	AGGC
7_+_91367286	13	AGGC
7_-_62185448	13	AGGC
1_-_9079601	12	AGGC
11_-_81227425	11	AGGC
3_+_68114083	11	AGGC
6_-_135826847	11	AGGC
2_-_70784811	10	AGGC
1_+_128176822	781	AGGG
14_+_77365024	196	AGGG
3_-_56043120	132	AGGG
X_-_103588172	88	AGGG
6_+_140536606	84	AGGG
4_-_36122181	57	AGGG
4_+_109737141	44	AGGG
12_+_4970404	41	AGGG
6_+_105678014	41	AGGG
6_+_65085726	34	AGGG
8_+_14454399	34	AGGG
7_+_123188393	32	AGGG
9_-_95497294	26	AGGG
17_+_55463884	25	AGGG
9_-_104196331	23	AGGG
4_-_70383419	23	AGGG
2_-_18298980	22	AGGG
X_-_103605310	21	AGGG
2_+_28846761	20	AGGG
5_-_9702032	18	AGGG
14_-_7310298	17	AGGG
13_+_63017857	17	AGGG
13_-_93177259	17	AGGG
3_-_26246847	16	AGGG
14_-_119081379	16	AGGG
1_-_162075787	15	AGGG
14_-_103264399	15	AGGG
11_+_72869177	14	AGGG
16_+_5886118	14	AGGG
10_+_14086014	13	AGGG
9_+_66424938	13	AGGG
X_-_47843695	13	AGGG
14_+_60831447	11	AGGG
X_+_69407511	11	AGGG
2_+_147052659	11	AGGG
5_-_5644561	11	AGGG
11_-_26036909	11	AGGG
1_+_86812486	11	AGGG
9_-_106713666	10	AGGG
7_-_75255477	10	AGGG
8_+_79662433	10	AGGG
7_-_62100437	417	AGAT
4_+_102281769	142	AGAT
4_-_36409784	120	AGAT
12_+_4978451	118	AGAT
10_+_50635031	110	AGAT
15_+_35491126	97	AGAT
7_-_61195040	91	AGAT
18_-_22951206	89	AGAT
16_+_6636583	84	AGAT
13_+_91525613	82	AGAT

Loci	#	Motif
1_-_124753317	71	AGAT
9_-_10168834	63	AGAT
2_-_39150415	53	AGAT
4_-_126392561	38	AGAT
5_+_34842732	30	AGAT
3_+_159080765	29	AGAT
12_+_61604964	29	AGAT
12_+_3476947	29	AGAT
6_+_111239501	27	AGAT
2_-_146459719	26	AGAT
7_-_61233587	22	AGAT
7_+_63461661	21	AGAT
9_+_86479623	20	AGAT
17_+_55463017	20	AGAT
9_+_94873127	19	AGAT
2_-_113816737	17	AGAT
2_+_166049646	17	AGAT
1_+_115689399	17	AGAT
2_+_164081521	17	AGAT
7_-_84083045	16	AGAT
14_-_124574599	16	AGAT
1_-_66807605	15	AGAT
5_-_67781092	15	AGAT
X_+_155806465	15	AGAT
1_-_119779051	15	AGAT
6_-_102514241	15	AGAT
5_+_81105225	15	AGAT
6_-_37004587	15	AGAT
2_+_50369944	14	AGAT
13_+_104369652	12	AGAT
6_+_143192859	12	AGAT
5_+_150750195	12	AGAT
3_-_56045343	12	AGAT
16_-_17703150	12	AGAT
14_+_15006634	11	AGAT
11_-_37224567	11	AGAT
X_+_155927605	11	AGAT
X_-_111595382	10	AGAT
8_+_85896667	10	AGAT
2_+_18094951	10	AGAT
13_+_18808321	10	AGAT
2_-_162295753	10	AGAT
8_+_65721127	10	AGAT
12_+_88853103	761	AGAA
4_+_135858840	318	AGAA
3_+_35808607	106	AGAA
9_+_111374733	100	AGAA
12_+_52938338	96	AGAA
10_+_39742689	95	AGAA
4_-_35967938	89	AGAA
7_-_61243933	77	AGAA
1_-_8996619	76	AGAA
11_-_33404200	42	AGAA
7_-_110456965	37	AGAA
18_-_62521491	36	AGAA
11_-_36957961	30	AGAA
2_-_66411458	29	AGAA
10_+_29196224	29	AGAA
9_-_4518942	28	AGAA
2_+_147034828	27	AGAA
5_-_107843897	23	AGAA
16_-_45718909	23	AGAA
3_-_56016556	21	AGAA
4_-_126375477	21	AGAA
5_-_107811403	16	AGAA
10_+_115390236	16	AGAA
12_+_53640695	14	AGAA
2_+_48989026	13	AGAA
4_-_36365137	12	AGAA
4_-_151579857	11	AGAA
2_+_96911007	11	AGAA
13_+_91538752	11	AGAA
19_+_55340712	11	AGAA
9_-_29011413	10	AGAA
3_+_116012185	10	AGAA
16_-_60483911	97	AGAC
3_-_148861290	73	AGAC
9_+_110546662	54	AGAC
9_+_66418367	46	AGAC
13_+_108685992	29	AGAC
19_+_36982810	29	AGAC

Loci	#	Motif
15_+_35665136	27	AGAC
8_+_112803002	24	AGAC
3_+_40870094	21	AGAC
5_+_71760382	21	AGAC
15_+_39536794	16	AGAC
X_+_155736382	15	AGAC
15_+_35616038	15	AGAC
3_-_30909135	15	AGAC
4_+_83437585	15	AGAC
4_+_42919790	15	AGAC
8_+_66703006	14	AGAC
10_+_85008655	14	AGAC
16_-_37230076	13	AGAC
8_+_45739954	11	AGAC
X_-_19232047	11	AGAC
5_-_86153367	10	AGAC
15_-_71681548	10	AGAC
1_-_154799699	135	AGAG
7_+_87586513	127	AGAG
12_+_116350140	124	AGAG
9_+_121729995	123	AGAG
2_-_17525490	76	AGAG
7_-_61197854	61	AGAG
4_+_98469218	51	AGAG
X_-_102408966	49	AGAG
9_-_78569945	48	AGAG
12_+_116353825	48	AGAG
6_+_55406755	32	AGAG
2_-_66371099	29	AGAG
X_+_155843739	28	AGAG
11_-_109982269	28	AGAG
15_+_91768084	27	AGAG
9_+_92578417	27	AGAG
12_+_16935149	23	AGAG
9_+_113841157	22	AGAG
18_+_49921499	16	AGAG
9_-_10658288	15	AGAG
14_-_105140017	15	AGAG
2_-_34714295	13	AGAG
9_-_16017279	13	AGAG
4_-_87133582	11	AGAG
17_+_55490910	10	AGAG
15_-_48039542	10	AGAG
6_+_110761323	10	AGAG
9_+_111347464	342	AGCA
11_-_102537147	259	AGCA
17_+_11303588	151	AGCA
3_-_158263393	146	AGCA
5_-_25415027	109	AGCA
12_+_89191151	68	AGCA
2_-_23541616	61	AGCA
9_-_121257422	59	AGCA
5_-_106921077	37	AGCA
18_+_36626002	35	AGCA
6_+_81933726	31	AGCA
8_+_45688296	25	AGCA
4_+_86780291	25	AGCA
1_+_128260829	25	AGCA
8_-_48311696	24	AGCA
1_+_66593459	20	AGCA
4_-_76101589	17	AGCA
1_-_54181363	13	AGCA
12_+_116342043	13	AGCA
8_-_32441050	12	AGCA
11_-_33304011	11	AGCA
10_+_58906407	11	AGCA
8_+_35779842	11	AGCA
19_+_57673405	11	AGCA
8_-_17392675	10	AGCA
4_+_49638923	10	AGCA
2_-_17462857	113	AGCC
1_-_9249411	90	AGCC
11_-_77605639	77	AGCC
5_-_49385846	63	AGCC
7_-_61221933	61	AGCC
12_+_89103786	22	AGCC
14_-_119083959	22	AGCC
12_+_52783308	21	AGCC
18_-_61390087	17	AGCC
11_+_29210900	15	AGCC
X_-_102618379	12	AGCC

Loci	#	Motif
2_-_94328885	12	AGCC
9_-_107838931	12	AGCC
2_+_65668739	11	AGCC
8_-_48837059	11	AGCC
17_+_79618976	10	AGCC
1_+_66638582	46	AGCG
16_+_5953417	17	AGCG
X_-_103591907	181	AGCT
4_-_24693635	79	AGCT
8_+_90973922	70	AGCT
9_+_27336115	66	AGCT
11_+_80399427	35	AGCT
3_+_60616059	35	AGCT
2_-_6849356	33	AGCT
6_-_122483223	32	AGCT
11_+_23682506	27	AGCT
7_-_78325452	24	AGCT
1_-_25752435	20	AGCT
7_-_61151350	18	AGCT
5_+_16284880	15	AGCT
9_-_79679432	15	AGCT
11_-_65743466	14	AGCT
2_+_67843337	13	AGCT
4_+_150431889	13	AGCT
14_-_62733851	13	AGCT
3_+_156920055	13	AGCT
7_-_61279511	12	AGCT
9_-_53213140	10	AGCT
2_+_20407682	10	AGCT
4_+_21811465	10	AGCT
3_+_68064042	10	AGCT
3_-_85121870	10	AGCT
14_-_103220149	714	AGGA
1_-_52204517	517	AGGA
13_-_103057916	200	AGGA
15_+_22817626	142	AGGA
4_+_104280468	125	AGGA
2_-_59983971	120	AGGA
7_-_61279760	109	AGGA
16_-_37156845	106	AGGA
10_-_5412911	102	AGGA
16_-_96008215	93	AGGA
18_+_49903412	86	AGGA
6_-_114031474	58	AGGA
4_-_58872098	57	AGGA
2_-_18295709	52	AGGA
15_-_53657526	45	AGGA
9_+_86510613	45	AGGA
4_+_33323653	43	AGGA
2_-_66293896	41	AGGA
10_+_90069848	38	AGGA
3_-_120797291	33	AGGA
1_-_84809033	31	AGGA
7_-_61181703	30	AGGA
16_-_37225135	28	AGGA
15_-_24990196	26	AGGA
9_-_65000178	23	AGGA
7_-_61129946	21	AGGA
3_+_95451656	21	AGGA
2_-_62762877	19	AGGA
6_+_104730653	19	AGGA
1_+_63456102	17	AGGA
1_+_86820839	17	AGGA
1_+_159258645	17	AGGA
10_+_63249952	16	AGGA
19_+_29535172	16	AGGA
10_+_98926312	15	AGGA
2_+_97555336	14	AGGA
9_-_10532185	14	AGGA
14_-_23604448	14	AGGA
10_+_14101625	14	AGGA
4_+_104346080	12	AGGA
4_-_11295505	11	AGGA
1_-_25827430	11	AGGA
1_+_86813653	11	AGGA
16_-_16469373	11	AGGA
Y_-_1216512	10	AGGA
1_-_132542239	10	AGGA
2_+_102751345	10	AGGA
4_-_36434683	10	AGGA
14_-_12776073	10	AGGA

Loci	#	Motif
3_-_26132804	177	AGTA
2_-_112860532	139	AGTA
1_-_52217299	47	AGTA
12_-_98242533	39	AGTA
1_-_155961191	30	AGTA
5_-_128770054	19	AGTA
5_-_107814716	18	AGTA
15_+_8182520	17	AGTA
6_-_35067813	17	AGTA
6_+_45826164	16	AGTA
4_-_3562147	15	AGTA
10_-_49650033	11	AGTA
7_+_113553294	11	AGTA
1_+_86812510	116	AGTC
12_+_29708833	64	AGTC
4_-_36739098	46	AGTC
12_+_16934239	30	AGTC
10_+_106835175	27	AGTC
3_+_29311798	24	AGTC
14_+_27699639	15	AGTC
10_-_5175929	14	AGTC
16_+_43282969	14	AGTC
14_+_64731659	13	AGTC
2_+_20438936	11	AGTC
5_+_109927200	11	AGTC
2_+_102751260	10	AGTC
10_+_106828875	102	AGTG
2_-_39082853	45	AGTG
15_+_81415845	33	AGTG
15_-_38011943	21	AGTG
4_-_19539181	20	AGTG
6_+_110703440	18	AGTG
10_+_101866675	16	AGTG
8_+_65670877	14	AGTG
1_+_192226324	14	AGTG
9_-_119290866	13	AGTG
14_-_39470887	13	AGTG
2_+_37555478	13	AGTG
15_+_97789651	13	AGTG
9_+_120947066	12	AGTG
14_-_119063306	12	AGTG
6_+_106077480	11	AGTG
15_+_39394199	10	AGTG
4_+_110428203	10	AGTG
18_+_38454313	10	AGTG
4_-_36279317	237	AGTT
16_-_37241245	215	AGTT
10_-_89084654	58	AGTT
2_-_62845339	56	AGTT
12_+_61768006	52	AGTT
14_-_57643490	49	AGTT
16_+_6636524	43	AGTT
6_+_33307200	42	AGTT
7_-_6725524	40	AGTT
3_-_127244269	34	AGTT
4_-_76388148	33	AGTT
X_+_50884576	31	AGTT
4_-_36339631	28	AGTT
X_-_21059804	27	AGTT
1_-_123433116	25	AGTT
1_+_140383258	24	AGTT
11_-_29819185	23	AGTT
4_-_123479060	20	AGTT
13_-_59801259	20	AGTT
14_-_103292665	20	AGTT
6_+_119906384	20	AGTT
6_-_37016750	19	AGTT
5_-_36630577	18	AGTT
7_+_96969500	16	AGTT
2_-_136318423	15	AGTT
2_+_49073840	15	AGTT
5_-_45727743	15	AGTT
5_+_81173433	15	AGTT
9_+_72298680	14	AGTT
12_-_3308193	12	AGTT
15_-_35104421	12	AGTT
8_+_23694298	11	AGTT
9_-_10791688	11	AGTT
4_+_33392713	11	AGTT
14_-_12694475	10	AGTT
8_-_8444936	10	AGTT

S2 Fig. The 405 AGNN RS site candidates. #, number of junction reads.
