# Supplementary material for: Recursive splicing is a rare event in the mouse brain
Source: PLoS One. 2022 Jan 28;17(1):e0263082. doi: 10.1371/journal.pone.0263082 (PMC8797253; doi:10.1371/journal.pone.0263082)
Supplement: S3 Fig — (A) Heatmap of phyloP score of RS sites and the flanking regions. (B) Boxplot of lengths of RS introns and introns transcribed in the mouse cortex. ***, P < 0.0001, one-tailed t-test. (C) Pie chart of locations of RS introns in host genes. (D) Heatmap of expression levels of RS genes in 22 mouse tissues. (E) The sequence motifs, nucleotide percentages, and 3’SS MaxEnt scores of the 20 RS sites. (F) Sequence logos of the 64 nt regions surrounding the 2640 non-RS AGGT sites and the 20 RS AGGT sites. (G) Schematic of the sequence base pairing between the AGGTAAGT motif and U1 snRNA. (H) Boxplots of the percentages of nucleotides in the 20 nt region upstream of the 2640 non-RS AGGT sites and the 20 RS AGGT sites. (I) Boxplot of MaxEnt 3’ splice site (3’SS) scores of the 20 RS AGGT sites and the 2640 non-RS AGGT sites. ***, P < 0.0001, one-tailed t-test. (PDF) [file pone.0263082.s003.pdf]

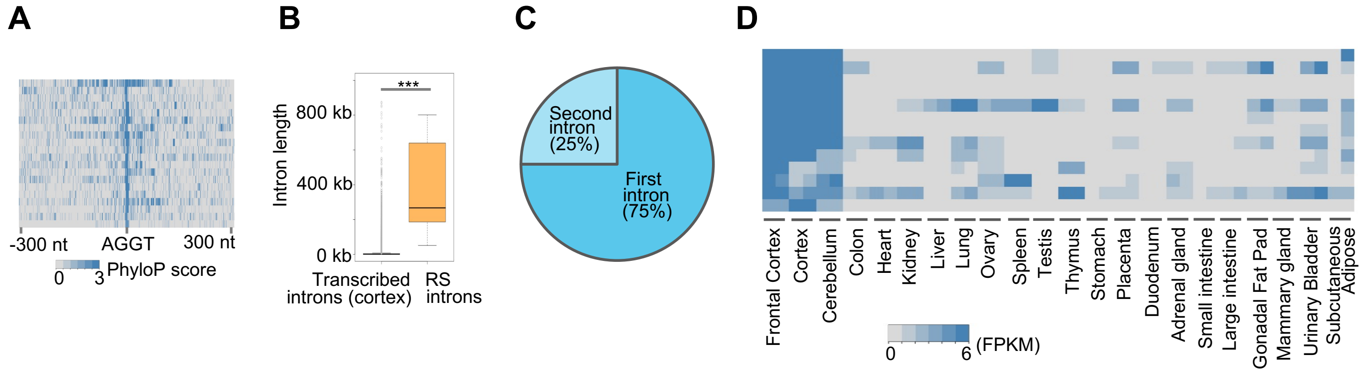

E

	Motif	Sequence of 20 nt upstream of AGGT	(T+C)%	T%	C%	G%	A%	3'SS MaxEnt score
chr16_+_40655810_*Lsamp*	AGGTAAGT	TTTCTTCTTTTCTCTCTTCC	1	0.65	0.35	0	0	14.81
chr9_+_47633924_*Cadm1*	AGGTAAGT	TTTTCTCTCCTCTTTCTTTT	1	0.7	0.3	0	0	13
chr13_+_109333510_*Pde4d*	AGGTAAGT	CTCATCTCTCTTTCTCTTTT	0.95	0.6	0.35	0	0.05	14.12
chr13_+_109614417_*Pde4d*	AGGTAAGT	TGCTTTTCTTTTTCTTTTTC	0.95	0.75	0.2	0.05	0	15.18
chr16_+_41206970_*Lsamp*	AGGTAAGT	TTTTTTTTCATTCTCTCCTT	0.95	0.7	0.25	0	0.05	12.87
chr16_+_40262266_*Lsamp*	AGGTAAGT	TTTTCTATTTTTTTTCTTCT	0.95	0.8	0.15	0	0.05	12.03
chr9_+_28029505_*Opcml*	AGGTAAGT	CCCTTTCTTTGTCTTTCCCT	0.95	0.55	0.4	0.05	0	13.71
chr10_+_69595048_*Ank3*	AGGTAAGT	TTTCTCTCTTTTTCTTTTAC	0.95	0.7	0.25	0	0.05	14.57
chr9_-_29674403_*Ntm*	AGGTAAGT	TCTCCCGTCTCTCTTTTTAT	0.9	0.55	0.35	0.05	0.05	12.55
chr16_+_40979498_*Lsamp*	AGGTAAGT	TGTTGTTTCTTTTTTCTTTC	0.9	0.75	0.15	0.1	0	13.26
chr16_-_74151682_*Robo2*	AGGTAAGT	TGGCTCTTCATTTCTTCTTC	0.85	0.55	0.3	0.1	0.05	10.73
chr2_+_179627792_*Cdh4*	AGGTAAGT	AAACCTTTCCTCTTATTCCT	0.8	0.45	0.35	0	0.2	10.26
chr11_-_33718264_*Kcnip1*	AGGTAAGT	ACTTCTGTGTTCTTTCTTGC	0.8	0.55	0.25	0.15	0.05	13.77
chr13_+_109550007_*Pde4d*	AGGTAAGT	TTTGTGTGTTTTGTTTTTTT	0.8	0.8	0	0.2	0	12.59
chr14_+_119537648_*Hs6st3*	AGGTAAGT	TGACTTCTGTCCCATATCTC	0.75	0.4	0.35	0.1	0.15	9.3
chr16_-_67142933_*Cadm2*	AGGTAAGC	TTTTGTTTCCTTTTATTTTT	0.9	0.8	0.1	0.05	0.05	11.73
chr16_-_67364247_*Cadm2*	AGGTGAGT	CTCCCCCTTCTTGTTTTTAT	0.9	0.55	0.35	0.05	0.05	12.33
chr9_-_49642257_*Ncam1*	AGGTAAGG	TATCTCACCTCACAACCAAA	0.6	0.2	0.4	0	0.4	3.15
chr9_-_49719364_*Ncam1*	AGGTAAGG	TCACTCTGCCCTGTTAAAAC	0.65	0.3	0.35	0.1	0.25	7.31
chrX_-_51473338_*Hs6st2*	AGGTAAGG	CTCTGGCCATCTGGACACTC	0.65	0.25	0.4	0.2	0.15	2.98

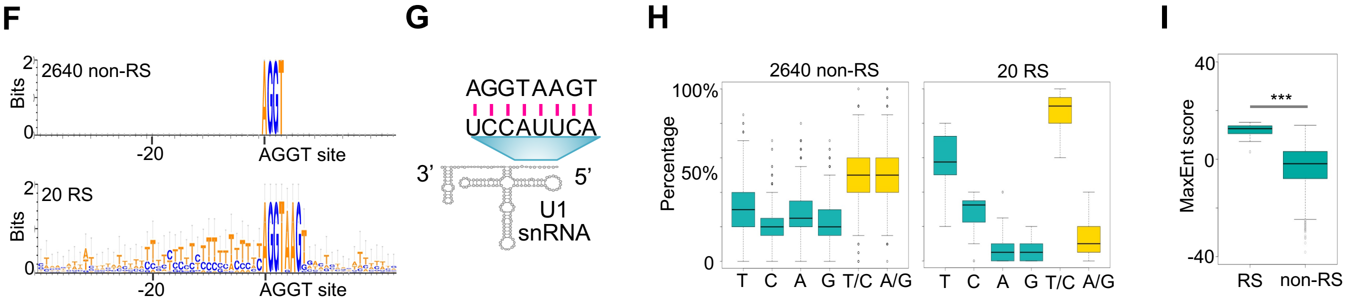

S3 Fig. Characteristics of RS sites. (A) Heatmap of phyloP score of RS sites and the flanking regions. (B) Boxplot of lengths of RS introns and introns transcribed in the mouse cortex. ***, *P* < 0.0001, one-tailed t-test. (C) Pie chart of locations of RS introns in host genes. (D) Heatmap of expression levels of RS genes in 22 mouse tissues. (E) The sequence motifs, nucleotide percentages, and 3'SS MaxEnt scores of the 20 RS sites. (F) Sequence logos of the 64 nt regions surrounding the 2640 non-RS AGGT sites and the 20 RS AGGT sites. (G) Schematic of the sequence base pairing between the AGGTAAGT motif and U1 snRNA. (H) Boxplots of the percentages of nucleotides in the 20 nt region upstream of the 2640 non-RS AGGT sites and the 20 RS AGGT sites. (I) Boxplot of MaxEnt 3' splice site (3'SS) scores of the 20 RS AGGT sites and the 2640 non-RS AGGT sites. ***, *P* < 0.0001, one-tailed t-test.
