# Supplementary material for: Recursive splicing is a rare event in the mouse brain
Source: PLoS One. 2022 Jan 28;17(1):e0263082. doi: 10.1371/journal.pone.0263082 (PMC8797253; doi:10.1371/journal.pone.0263082)
Supplement: S4 Fig — (A) The genomic loci, numbers of junction reads, 5’SS sequences, and 5’SS MaxEnt scores of RS exons. (B) Schematic of the pipeline utilizing nuclear total RNA-seq data to identify RS-like cryptic exons. (C) The genomic loci, numbers of junction reads, 5’SS sequences, and 5’SS MaxEnt scores of RS-like cryptic exons. (D) Sequencing profile at Magi1 locus. Green arrows indicate the putative RS AGGT loci. (PDF) [file pone.0263082.s004.pdf]

A

RS exon	Strand	Gene	Number of Junction reads		Upstream exon ending	r5'SS		Down 5'SS	
			Up	Down		Sequence	MaxEnt score	Sequence	MaxEnt score
chr16:67364176-67364248	-	*Cadm2*	317	22	chr16:67620171	AAGGTGAGT	10.47	TGGGTAAGT	10.24
chr16:67142893-67142934	-	*Cadm2*	219	22	chr16:67620171	AAGGTAAGC	10.22	AAGGTAAAA	8.38
chr16:41206972-41207028	+	*Lsamp*	100	5	chr16:40786060	CAGGTAAGT	10.86	GAGGCAAGT	3.32
chr9:29674281-29674404	-	*Ntm*	475	5	chr9:29962845	AAGGTAAGT	11	CAGGTAGGT	10.28
chr16:40262268-40262469	+	*Lsamp*	161	5	chr16:39984727	AAGGTAAGT	11	AAGGTAAGT	11
chr10:69595050-69595128	+	*Ank3*	173	4	chr10:69534284	AAGGTAAGT	11	AAGGTTCGT	8.44
chr16:40655812-40655859	+	*Lsamp*	79	4	chr16:39984727	AAGGTAAGT	10.86	GTGGTAAGT	10.36
chr9:47633926-47634076	+	*Cadm1*	62	4	chr9:47530505	CAGGTAAGT	10.86	CAGGTAATT	8.55
chr16:40979500-40979631	+	*Lsamp*	112	3	chr16:40786060	CAGGTAAGT	11	CAGGTGCTT	4.83
chr9:28029507-28029642	+	*Opcml*	334	1	chr9:27791446	CAGGTAAGT	10.86	AAGGTATGG	9.26
chr14:119537650-119537790	+	*Hs6st3*	202	0	chr14:119139118	CAGGTAAGT	10.86	TTGGTGACA	-1.41
chr13:109550009-109550238	+	*Pde4d*	42	0	chr13:109442353	ACGGTAAGT	11.81	CAAGTACAA	-1.09
chr13:109614419-109614568	+	*Pde4d*	33	0	chr13:109442353	ACGGTAAGT	11.81	TTGGTTTGG	-1.65
chr2:179627794-179627953	+	*Cdh4*	25	0	chr2:179444811	AAGGTAAGT	11	TTGGTCTGT	0.67
chr9:49642143-49642258	-	*Ncam1*	135	0	chr9:49798678	CAGGTAAGG	11.08	AAAGTGTGA	-1.44
chr13:109333512-109333719	+	*Pde4d*	128	0	chr13:109117080	ACGGTAAGT	11.81	AAAGTTGGA	-3.21
chr16:74151336-74151683	-	*Robo2*	97	0	chr16:74352551	CATGTAAGT	8.31	TTGGTTATA	-5.68
chr9:49719316-49719365	-	*Ncam1*	83	0	chr9:49798678	CAGGTAAGG	11.08	AAGGTTTCT	0.28
chrX:51473121-51473339	-	*Hs6st2*	26	0	chrX:51679890	CAGGTAAGG	11.08	ATGGTTTTA	-5.54
chr11:33718032-33718265	-	*Kcnip1*	8	0	chr11:33843130	AAGGTAAGT	11	CTGGTGGCC	-2.74

B

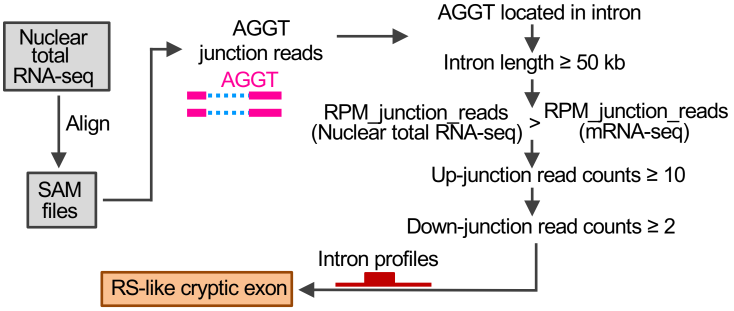

D

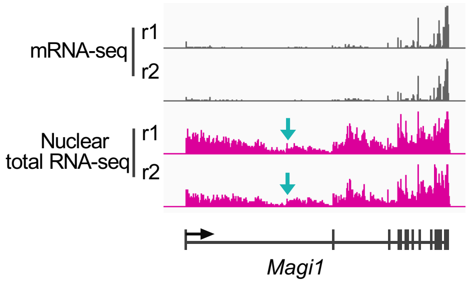

C

RS-like cryptic exon	Strand	Gene	Number of Junction reads		Upstream exon ending	r5'SS		Down 5'SS	
			Up	Down		Sequence	MaxEnt score	Sequence	MaxEnt score
chr2:17526050-17526130	-	*Nebl*	51	154	chr2:17529910	AATGTGGAA	-11.32	ATGGTAAGT	11.01
chr2:97522122-97522199	+	*Lrrc4c*	59	68	chr2:97468420	ACAGTGGAT	-7.51	AAGGTTGGA	5.64
chr7:61241310-61241445	-	*A230006K03Rik*	91	51	chr7:61282776	AAGGTGTTT	2.06	CAGGTTTGT	7.44
chr12:46766290-46766391	-	*Nova1*	61	50	chr12:46816886	CAGGTGCTG	4.87	ACTGTAAGT	8.59
chr5:57785821-57785880	+	*Pcdh7*	62	41	chr5:57722278	CAGGTGGAT	3.56	AATGTATGT	5.87
chr1:123595259-123595324	-	*Dpp10*	25	31	chr1:123650288	CAGGTGCTT	4.83	GAGGTAACA	8.1
chr1:124638974-124639139	-	*Dpp10*	43	34	chr1:124845719	AAGGTGTCT	2.57	CAGGTACGG	10.88
chr12:44527672-44527848	+	*Nrcam*	55	31	chr12:44456702	AAGGTTCCT	0.89	TTGGTATAT	1.75
chr3:158361404-158361562	-	*Lrrc7*	27	31	chr3:158413130	CAAGTCTCC	-11.96	AAGGTGAGA	8.68
chr7:75224697-75224831	-	*Sv2b*	43	25	chr7:75308205	AAGGTCTGG	3.93	CTGGTCAGT	6.6
chr2:136200589-136200635	-	*Pak7*	23	23	chr2:136269323	AAAGTTTCT	-9	AAGGTAAAA	8.38
chrX:77584794-77584859	+	*Tbl1x*	27	20	chrX:77511269	GAGGTAATT	8.88	TGAGTAAGT	8.82
chr3:159150266-159150351	+	*Gm20752*	21	19	chr3:159090393	AAGGTACAG	8.04	TTAGTAAGT	7.79
chr2:7282441-7282578	-	*Celf2*	24	18	chr2:7395858	CTGGTCTTT	-3.78	AAAGTAAGT	9.72
chr7:64506937-64507022	+	*Apba2*	17	17	chr7:64502253	CAGGTCTCC	0.85	GAGGTAGGA	8.24
chr16:6158060-6158206	+	*Rbfox1*	12	14	chr16:5885864	TAGGTGCAG	0.29	AAAGTAAGT	9.72
chr6:94050641-94050766	-	*Magi1*	41	13	chr6:94283010	AAGGTAAGG	10.51	GTTGTAAGT	8.3
chr3:29170414-29170593	+	*Egfem1*	18	13	chr3:29153601	ATGGTTAAA	-2.37	AAGGTAAAG	9.06
chr8:54881710-54881841	-	*Wdr17*	38	12	chr8:54886907	AAGGTGCTT	4.72	CAGGTACCA	7.88
chr8:50161927-50162063	-	*Gm45341*	10	11	chr8:50415134	AACGTGTTC	-7.75	CTGGTGAGT	10.1
chr7:96232099-96232201	+	*Tenm4*	15	9	chr7:96211990	ACTGTGAAG	-5.79	ACTGTAAGG	5.19
chr7:84005117-84005995	-	*Cemip*	23	2	chr7:84086258	GAGGTGACA	3.85	CAGGTCCGA	4.4

S4 Fig. RS exons and RS-like cryptic exons. (A) The genomic loci, numbers of junction reads, 5'SS sequences, and 5'SS MaxEnt scores of RS exons. (B) Schematic of the pipeline utilizing nuclear total RNA-seq data to identify RS-like cryptic exons. (C) The genomic loci, numbers of junction reads, 5'SS sequences, and 5'SS MaxEnt scores of RS-like cryptic exons. (D) Sequencing profile at *Magi1* locus. Green arrows indicate the putative RS AGGT loci.
