# Supplementary material for: Recursive splicing is a rare event in the mouse brain
Source: PLoS One. 2022 Jan 28;17(1):e0263082. doi: 10.1371/journal.pone.0263082 (PMC8797253; doi:10.1371/journal.pone.0263082)
Supplement: S5 Fig — Cadm2 RS1: chr16:67364249. Cadm2 RS2: chr16:67142935. Lsamp RS1: chr16:40262266. Lsamp RS2: chr16:40655810. Lsamp RS3: chr16:40979498. (PDF) [file pone.0263082.s005.pdf]

Primer name	Sequence (5' -> 3')
Cadm2 RS1 Forward Upstream-exon	GATCCTTCCCCAGACCCTTA
Cadm2 RS1 Reverse Downstream-exon	TGAGCTGGATTTGACCACTG
Cadm2 RS1 Forward RS-exon	GCTCCTTTATTAATGTCCTGTGG
Cadm2 RS1 Reverse RS-exon	CCACAGGACATTAATAAAGGAGC
Cadm2 RS1 Reverse Negative-control intron	CTATAAAAACAAGAAGGGGGA
Cadm2 RS2 Forward Upstream-exon	GATCCTTCCCCAGACCCTTA
Cadm2 RS2 Reverse Downstream-exon	TGAGCTGGATTTGACCACTG
Cadm2 RS2 Forward RS-exon	GTAAGCACATTAGTCATTCCTGT
Cadm2 RS2 Reverse RS-exon	TTGATGCAGTCTGATTATACAGG
Cadm2 RS2 Reverse Negative-control intron	CTAAAAATAAAAGGAAACAA
HS6ST3 Forward Upstream-exon	CTGGACCGAGCTCACCAAC
HS6ST3 Reverse Downstream-exon	CGTCGCACATATGGAGAGAGGT
HS6ST3 Forward RS-exon	CCTGTGTTGGAGTGACATACGA
HS6ST3 Reverse RS-exon	TCGTATGTCACTCCAACACAGG
HS6ST3 Reverse Negative-control intron	CTGAGATATGGGACAGAAGT
Ncam1 Forward Upstream-exon	GTCTGCTCCTCGGTCCA
Ncam1 Reverse Downstream-exon	CTCCTTGGCTGGGAACAATA
Ncam1 Forward RS-exon	GTAAGGAACTGCCAATCACG
Ncam1 Reverse RS-exon	CACGTGATTGGCAGTTCC
Ncam1 Reverse Negative-control intron	CTTTTGGTTGTGAGGTGAGAT
Lsamp Forward Upstream-exon	TGAGGGAAACCAAGGGAAGC
Lsamp Reverse Downstream-exon	CGCAAGCCCTACCTGAGGAT
Lsamp RS1 Reverse RS-exon	ACTCATCATTGCTGTGGGTGG
Lsamp RS2 Reverse RS-exon	TTTCTAAAGGACAGTCTGGGGA
Lsamp RS3 Reverse RS-exon	CTATGAGAATTGCCACCTGTGC
Lsamp Reverse Negative-control intron	TCTCTCCAGACTGATGGAGGAT

Primer name	Sequence (5' -> 3')
Lrr4c Forward Upstream-exon	TTGGTGTCAAGACCGTGCATTC
Lrr4c Reverse Downstream-exon	AGCCAACAGCACCACAAGC
Lrr4c Forward Cryptic-exon	CCATGGCCGGAATGGAATAAG
Lrr4c Reverse Cryptic-exon	CTTATTCCATTCCGGCCATGG
Lrr4c Reverse Negative-control intron	CTACAAATCCAAGAAGGGGA
Nova1 Forward Upstream-exon	AACTGGAGCCACTATCAAGCTG
Nova1 Reverse Downstream-exon	GATGCGATCAGGATTAACGGTG
Nova1 Forward Cryptic-exon	TGCCAGCGGCAGAAGAGAT
Nova1 Reverse Cryptic-exon	TGTGGTACTCCTAGTCAGTGTC
Nova1 Reverse Negative-control intron	CTAGGGGAAAGCGCGCTGTT
Magi1 Forward Upstream-exon	TCCAGAAGAAGAACCACTGGAC
Magi1 Reverse Downstream-exon	CATGGCGGTAGAGGTTGTC
Magi1 Forward Cryptic-exon	CAGATGCTGTCTTCCTGTGTTC
Magi1 Reverse Cryptic-exon	GTAGGCGAGCATATTGTGAACG
Magi1 Reverse Negative-control intron	CTGAAAAACATAAACACATA

S5 Fig. RT-PCR primer sequences for the RS sites (top table) and RS-like cryptic exons (bottom table). *Cadm2* RS1: chr16:67364249. *Cadm2* RS2: chr16:67142935. *Lsamp* RS1: chr16:40262266. *Lsamp* RS2: chr16:40655810. *Lsamp* RS3: chr16:40979498.
