# Supplementary figures and images for: Recursive splicing is a rare event in the mouse brain
Source: PLoS One. 2022 Jan 28;17(1):e0263082. doi: 10.1371/journal.pone.0263082 (PMC8797253; doi:10.1371/journal.pone.0263082)

*Hs6st3*

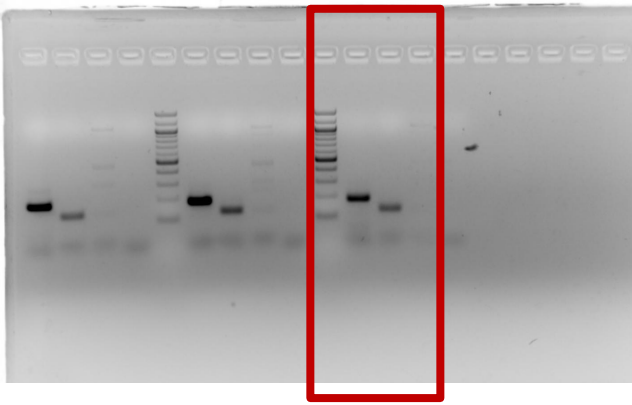

*Cadm2 (chr16:67364249)*

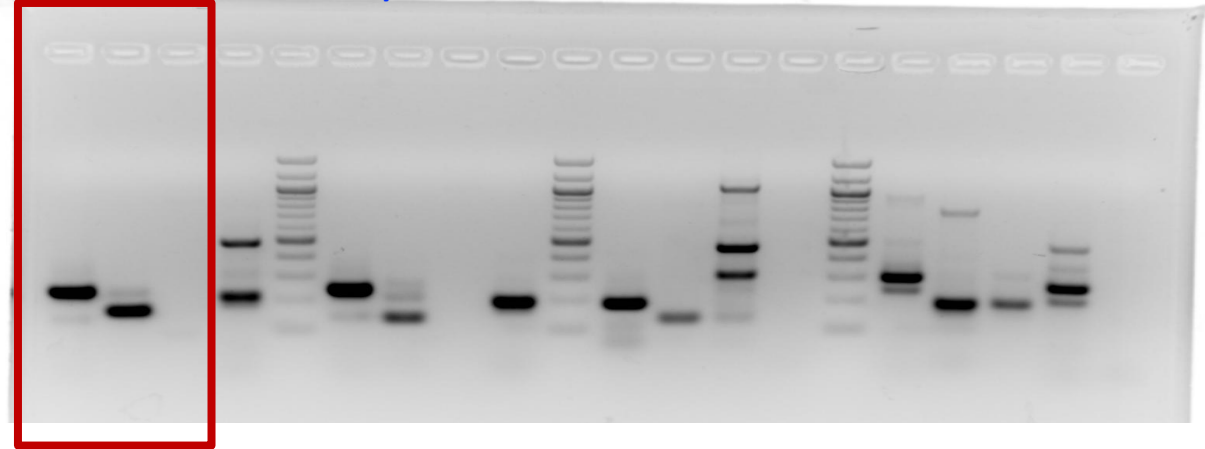

*Cadm2 (chr16:67142935)*

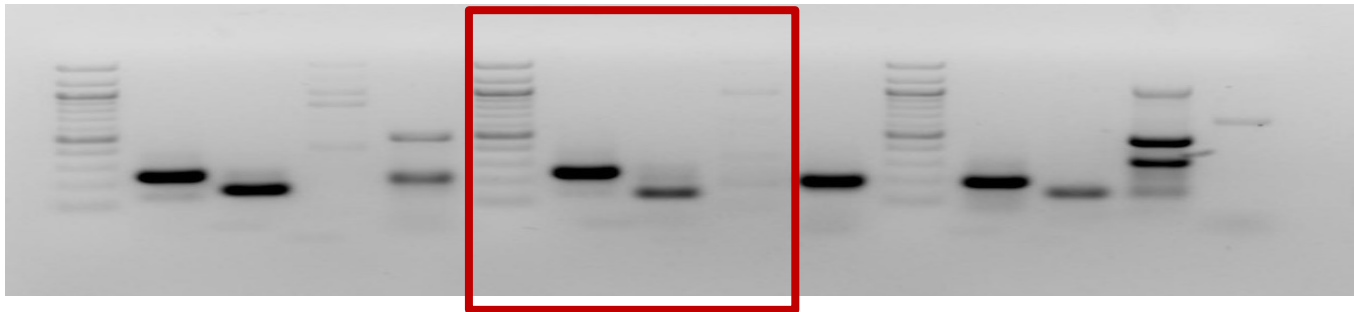

*Lsmp*

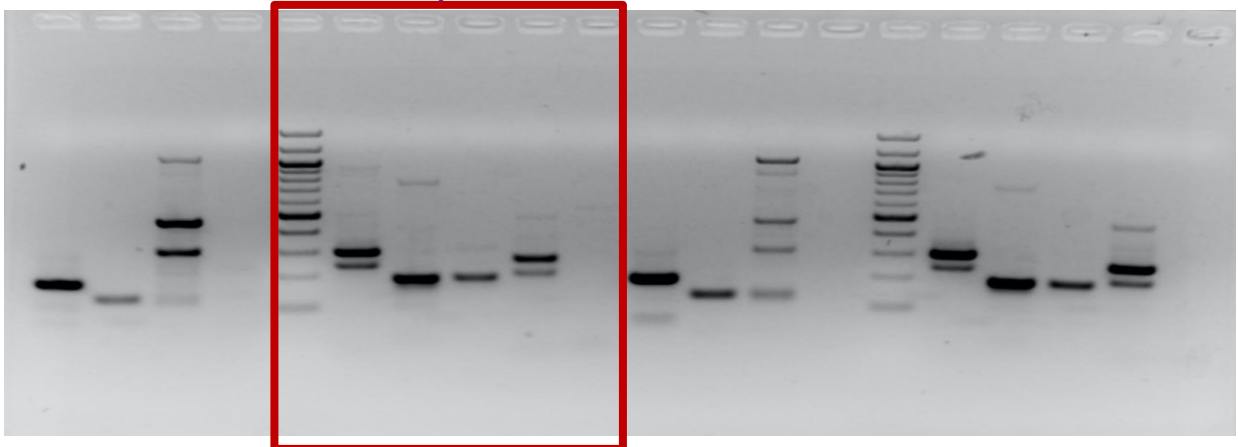

S6 Fig. The full-length gels for plots in Fig 2.

Supplement: S6 Fig — (PDF) [file pone.0263082.s006.pdf]

*Cadm2* (chr16:67364249)

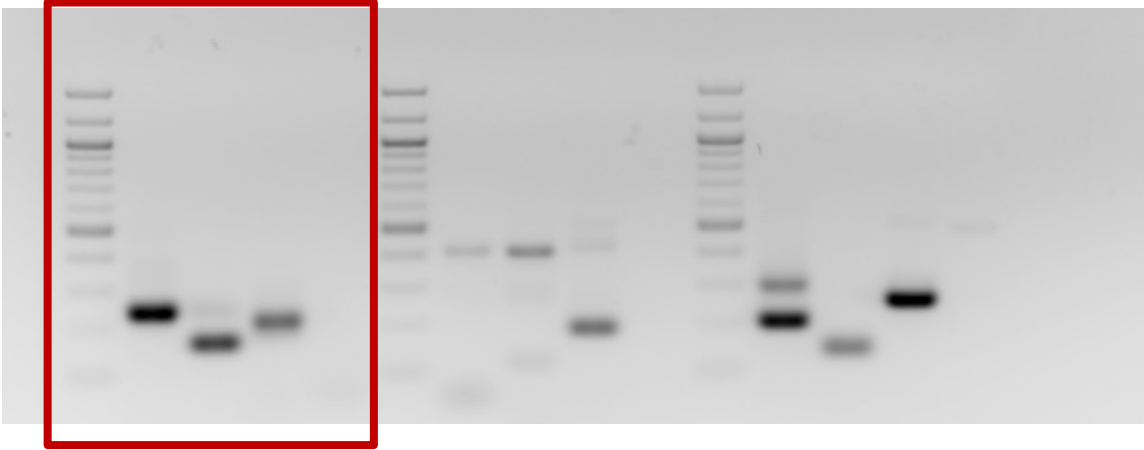

*Cadm2* (chr16:67142935)

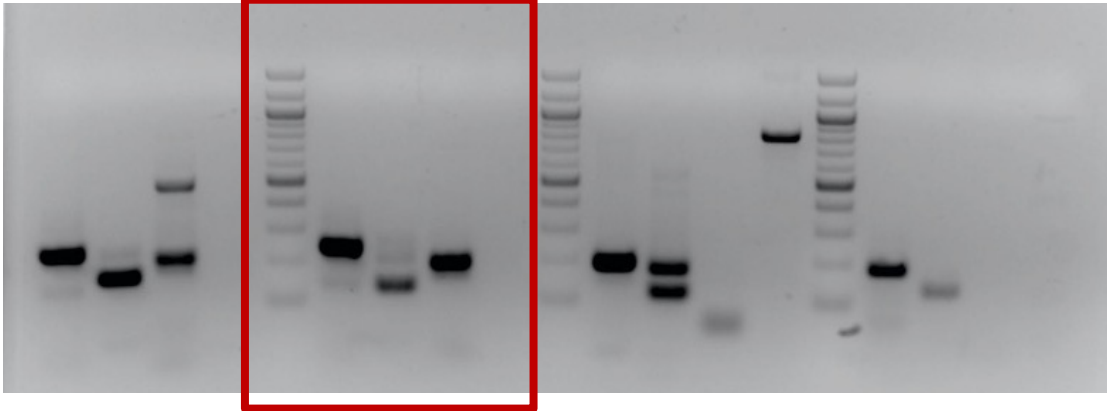

*Hs6st3*

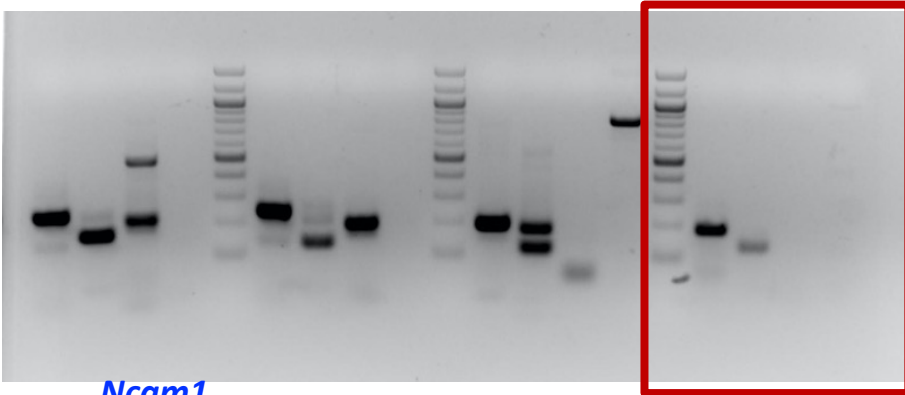

*Ncam1*

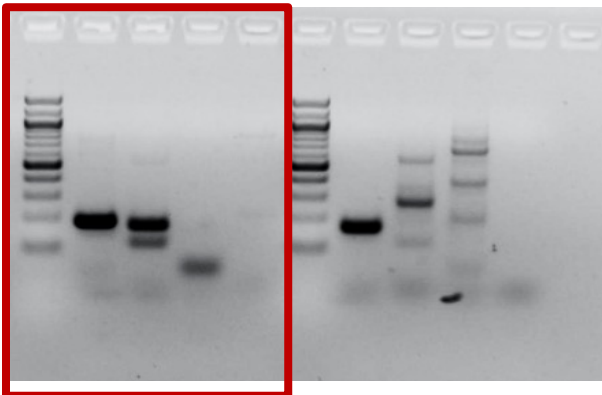

S7 Fig. The full-length gels for plots in Fig 4.

Supplement: S7 Fig — (PDF) [file pone.0263082.s007.pdf]

*Lrrc4c*

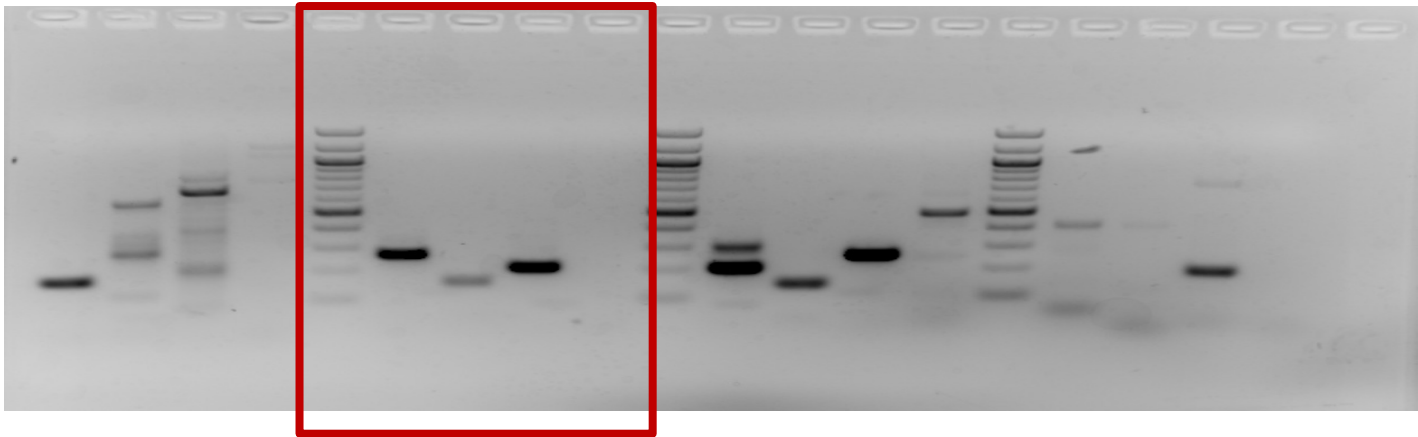

*Magi1*

*Nova1*

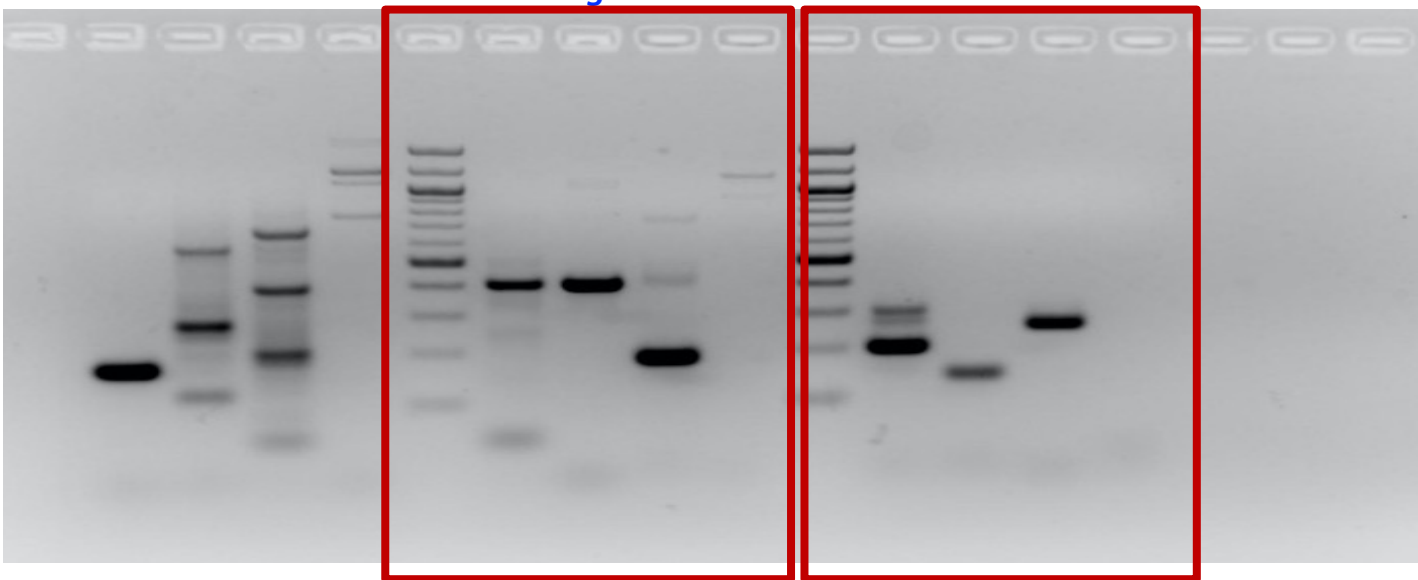

S8 Fig. The full-length gels for plots in Fig 5.

Supplement: S8 Fig — (PDF) [file pone.0263082.s008.pdf]
